# Structural and myocardial dysfunction in heart failure beyond ejection fraction

**DOI:** 10.1007/s10741-019-09828-8

**Published:** 2019-07-17

**Authors:** Paolo Severino, Viviana Maestrini, Marco Valerio Mariani, Lucia Ilaria Birtolo, Rossana Scarpati, Massimo Mancone, Francesco Fedele

**Affiliations:** Department of Cardiovascular, Respiratory, Nephrology, Anesthesiology and Geriatric Sciences, Sapienza University of Rome, Rome, Italy

**Keywords:** Heart failure, Ejection fraction, Imaging, Classification

## Abstract

Heart failure is a multifaceted syndrome addressing for a high rate of death among the general population. The common approach to this disease has been always based on the evaluation of the left ventricular ejection fraction by two-dimensional echocardiography with Simpson’s method. Mounting evidences have demonstrated the pitfalls of this method and have suggested that the management of heart failure requires a deep knowledge of the pathophysiological insights of the disease and cannot rely only on the evaluation of the left ventricular ejection fraction. Several advanced imaging technologies overwhelm the evaluation of ejection fraction and could provide a better understanding of the myocardial abnormalities underlying heart failure. Considering the limitation of left ventricular ejection fraction and the systemic involvement of heart failure, classifications of heart failure based on ejection fraction should be substituted with a comprehensive “staging” of multiorgan damage, not only considering the heart but also the lungs, kidneys, and liver, such as the HLM staging system. Such a holistic approach based on the HLM staging system and multimodality imaging can provide a global assessment of patient features allowing for targeted therapies and better heart failure management.

## Introduction

Heart failure (HF) is a complex clinical syndrome related to a wide spectrum of left ventricular function abnormalities. Nowadays, HF is one of the most common causes of hospitalization and death with great impact on social and economic resources [1]. During the past decades, physicians endeavored to classify HF in order to improve the understanding of this multifaceted syndrome and best serve the needs of patients.

Starting from the assumption of HF as a mechanical dysfunction of the heart, the measure of left ventricular (LV) function as the fraction of the LV end-diastolic volume ejected per beat was considered the best parameter for the detection and management of heart abnormalities. Firstly, Folse and Braunwald used a radioisotope indicator dilution technique [2] to estimate LV function; later, Bartle et al. [3] assessed angiographically LV function and the term left ventricular ejection fraction (LVEF) was coined. More recently, the development of two-dimensional echocardiography allowed the use of LVEF as a primary measure of left ventricular function and heralded the widespread adoption of LVEF-based classification of HF.

In 2012, the European Society of Cardiology HF guidelines [4] proposed a classification for HF basing on the evaluation of LVEF, dividing HF patients into two different groups: patients with typical symptoms of HF and LVEF < 35%, identified as the group with HF reduced EF (HFrEF) or “systolic HF,” and patients with clinical features of HF and LVEF > 40–45%, identified as the group with the so-called HF preserved EF (HFpEF) or “diastolic HF.” In 2016 HF ESC Guidelines [5], a third class was added covering the gray area between HFrEF and HFpEF: the HF mid-range EF (HFmrEF), defined as HF with EF in the range of 40–49%.

Although LVEF calculated through two-dimensional echocardiography is the mainstay for the evaluation of LV function and is used to classify HF patients, it has some important limitations such as limited test-retest reliability due to inter- and intraobserver variability, preload and afterload dependence that leads to loss of reproducibility, and poor image quality, resulting in foreshortened ventricles [6, 7]. Moreover, two-dimensional echocardiography requires geometric assumption on the LV shape to estimate LV volumes based on linear or two-dimensional measurements, possibly leading to errors [8].

Beyond the abovementioned limitations of LVEF, the ESC classification for HF has raised some criticisms because it considered a speculative distinction not helpful for daily clinical practice. A classification should provide detailed characterization of a patient and prognostic discrimination and should allow for therapies targeted on each patient’s phenotype. Furthermore, classifying should imply mechanistic insights, grouping patients based on critical pathophysiological abnormalities [9].

Conversely, LVEF is not related to any specific clinical feature or mechanistic insights; thus, LVEF-based HF classification may result inappropriate in providing pathophysiological distinctions. In fact, patients with HFpEF often have subclinical systolic impairment detected by speckle tracking echocardiography as reduction of global longitudinal strain (GLS) [10], whereas marked abnormalities in diastolic filling are typically present in HFrEF. Thus, EF cannot arbitrarily distinguish diastolic and systolic dysfunction, by itself.

Additionally, the introduction of a third distinct group of patients with an LVEF of 40 to 49%, the HFmrEF group, seems to be inappropriate and results in misleading and confusing findings. This group does not really exist in daily practice as a distinct class of HF patients because it has no distinguishable features and appears to be a transitory phase between two extremities of the wide spectrum of HF manifestations [11]. Though the HFmrEF group has fostered new studies, patients with mid-range LVEF have not been sufficiently characterized yet and the underlying pathophysiology, therapeutic responses, and prognosis remain unclear. What is clear is that parameters other than LVEF should be used to shed light on this clinical phenotype and its features.

Lastly, although a good predictor of cardiac events when LVEF is below 45%, the LVEF-based HF classification has shown a limited prognostic value in predicting the risk of all-cause morbidity and mortality in patients with EF > 45% [12], thus resulting in an unexpected high rate of adverse outcomes among patients with HFpEF. Moreover, several evidences pointed out the limited value of LVEF as a parameter to target therapies and ICD implantation for the primary prevention of SCD [13]. These data suggest that factors beyond EF should be used to define prognosis and effective treatments in HF patients.

Overall, several pitfalls reduce the reliability of LVEF measured through two-dimensional echocardiography and make the LVEF-based classification of HF less useful for patients and clinicians. The aforementioned limitations of the ESC classification system warrant the use of a more comprehensive assessment of HF and LV function, beyond the LVEF, using advanced imaging technologies such as three-dimensional echocardiography, longitudinal strain by speckle tracking echocardiography, and cardiovascular magnetic resonance (CMR) with evaluation of late gadolinium enhancement (LGE). These techniques could allow better definition of pathophysiology, management, and prognosis of HF rather than LVEF alone.

## History of heart failure/cardiomyopathy classifications proposed by American and European societies

**Fig. 1:**
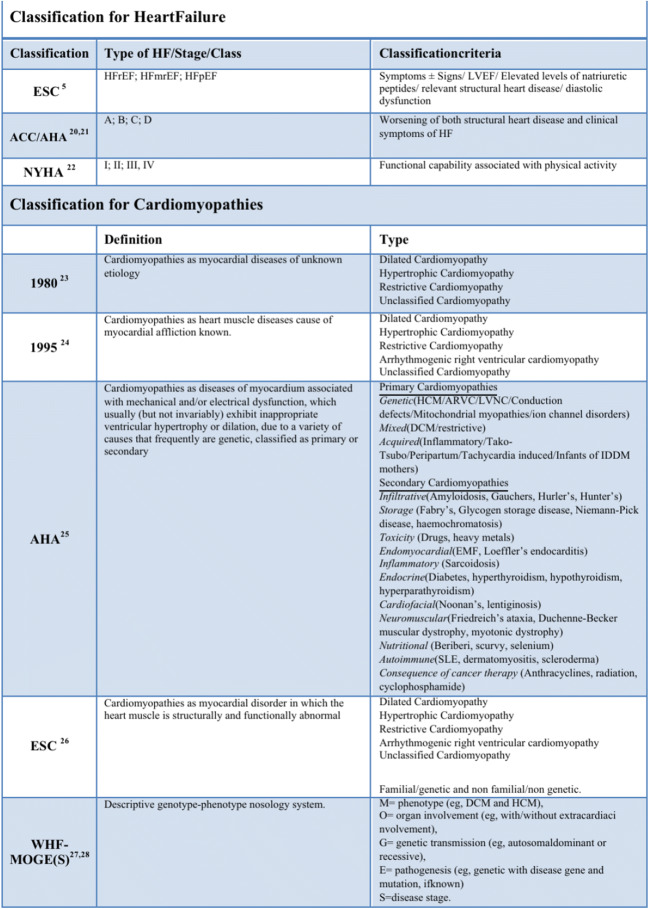
Classifications of heart failure and cardiomyopathies. AHA, American Heart Association; ARVC, arrhythmogenic right ventricular cardiomyopathy/dysplasia; DCM, dilated cardiomyopathy; EMF, endomyocardial fibrosis; ESC, European Society of Cardiology; HCM, hypertrophic cardiomyopathy; HF, heart failure; LVNC, left ventricular non-compaction

From the 1990s, HF patients began to be selected based on LVEF. “HFpEF” replaced the old “diastolic” heart failure, and HFrEF replaced the old “systolic” heart failure because diastolic dysfunction of the left ventricle may also characterize HFrEF [14] and subtle abnormalities of systolic function may be also found in patients with HFpEF. The range of “normality” of LVEF in heart failure has been long debated [15, 16]. According to the European Study Group on Diastolic Heart Failure [17], diagnostic criteria for HFpEF were (a) clinical symptoms and signs, (b) normal or mildly reduced LV systolic function (LVEF > 50% and LVEDVI < 97 mL/m^2^), and (c) diastolic dysfunction. In 2012, the European Society of Cardiology HF guidelines [16] proposed a classification for HF basing on the evaluation of LVEF, dividing HF patients into two different groups: patients with typical symptoms of HF and LVEF < 35%, identified as having HF reduced EF (HFrEF) or “systolic HF,” and patients with clinical features of HF and LVEF > 40–45%, identified as having the so-called HF preserved EF (HFpEF) or “diastolic HF” [18]. In 2016 HF ESC Guidelines (Fig. 1), a third class was added covering the gray area between HFrEF and HFpEF: the HF mid-range EF (HFmrEF), defined as HF with EF in the range of 40–49%. Further studies are warranted to clarify its risk factors, demographics, comorbidities, and pathophysiological processes and whether its treatments should be similar to those used for HFrEF [19]. Two prominent classification systems for HF are those of the American College of Cardiology and the American Heart Association (ACC/AHA) and of the New York Heart Association (NYHA). The stages of the ACC/AHA system (A to D) are based on worsening of both structural heart disease and clinical symptoms of HF. The NYHA designations (classes I to IV) are based on the functional capability associated with physical activity (Fig. 1) [20–22]. In the complexity of HF syndrome, identification of pathophysiology and etiology is the only way to define a correct diagnosis. Physicians must be reminded that management of HF is not just taking care of its symptoms. It is mandatory to decipher the mechanisms underlying HF that is a multiorgan syndrome. The aim of assisting clinicians should be to look beyond schematic diagnostic labels in order to achieve more specific diagnosis. In this regard, it is important to mention also cardiomyopathy (CMP) classifications (Fig. 1) that represent an important cause of heart failure. WHO/ISFC Task Force 1980 [23] defined CMP as myocardial diseases of unknown etiology and identified dilated, hypertrophic, restrictive, and unclassified CMP. The arrhythmogenic right ventricular cardiomyopathy/dysplasia (ARVC) was added later. WHO/ISFC Task Force 1995 [24] defined CMP as heart muscle diseases caused by known myocardial affliction. In both WHO/ISFC documents, the approach to CMP was based mainly on anatomical and morphological criteria and on a clinical phenotype. In the following years, molecular genetics was introduced. In 2006, the American Heart Association proposed the following definition [25]: “Cardiomyopathies are a heterogeneous group of diseases of the myocardium associated with mechanical and/or electric dysfunction that usually (but not invariably) exhibit inappropriate ventricular hypertrophy or dilatation and are due to a variety of causes that frequently are genetic, classified as primary or secondary. AHA presented first visionary attempt to classify primary cardiomyopathy by genetic origin (genetic, acquired, or mixed). Cardiomyopathies either are confined to the heart or are part of generalized systemic disorders.” In a departure, this panel added channelopathies to the CMP. ESC [26] defined CMP as a myocardial disorder in which the heart muscle is structurally and functionally abnormal. ESC classified dilated, hypertrophic, restrictive, arrhythmogenic right ventricular, or unclassified cardiomyopathy subtypes as familial/genetic and non-familial/non-genetic. The importance of phenotype preceding genetic classification for clinical practice was maintained. Later, oncologists have developed the so-called TNM system for cancer that has been successfully used for many years. In 2013, Arbustini et al. [27] proposed the MOGE(S) classification for cardiomyopathy, endorsed by the World Heart Federation. M refers to the phenotype (e.g., DCM and HCM), O refers to organ involvement (e.g., with/without extra cardiac involvement), G refers to genetic transmission (e.g., autosomal dominant or recessive), E refers to pathogenesis (e.g., genetic with disease gene and mutation, if known), and S refers to disease stage. Each letter in the MOGE(S) classification has well-defined subscripts, which provide details [28]. The MOGE(S) classification has several advantages with regard to simultaneous maximal description of disease from clinical and genetic points. However, this classification does not fulfill the diagnostic criteria of cardiomyopathies in several clinical situations and may not be always applied in clinical practice, because of the lack of genetic testing in many clinical centers. Additionally, the classification based on systematically genetic testing and monitoring may cause overdiagnostic states without clinically evident signs of cardiomyopathies and absence of clinical phenotype.

Further genetic research and development of multicenter registries are needed to clarify the clinical advantages and to make MOGE(S) classification of CMP more practical.

### Beyond ejection fraction and its limits: advanced imaging

Left ventricle global function is usually assessed by LVEF expressed as a percent value and calculated from estimations of LV volumes. The ideal imaging technique to assess LV cardiac function would be widely available, cheap, fast, with no need of ionizing radiation or contrast administration, and able to provide accurate and reproducible measurements. There is no imaging test meeting all of these characteristics. Echocardiography has some advantages as it is non-invasive, widely available, portable and relatively inexpensive. The biplane method of disks (modified Simpson’s rule) is the currently recommended two-dimensional (2DE) method [29]; however, it carries well-known technical limitations. Echocardiography is a non-tomographic technique: the 2DE LVEF estimation is based on measurements of areas in two single planes requiring inference to estimate the LV shape in order to calculate the three-dimensional volumes. This method can potentially cause errors due to LV foreshortened, LV cavity geometrical assumption and inadequate endocardial definition because of low quality images.

Three-dimensional echocardiography (3DE) provides volumes with minimal post-processing and overcomes some of the 2DE limitations such as the geometrical assumption. 3DE-based LVEF measurements are more accurate and reproducible with the closest approximation to CMR-derived measurements [30–33], and it is the recommended technique when acoustic window is adequate for analysis and the echo-laboratory has enough experience [29]. However, also 3DE has some disadvantages. 3DE quality depends on patient factors (breath-hold, hearth rhythm, acoustic window) and needs a deep knowledge of the echo settings during acquisition in order to obtain the best image possible [34]. These limitations are responsible for the loss of reproducibility and can result in a 5- to 7-point variability [8].

Independent of the technology used, LVEF has intrinsic limitations: LVEF is not an early marker of disease being normal even in the case of impaired heart and is also affected by loading condition. Accordingly, other parameters beyond LVEF have been studied to assess systolic function.

The assessment of myocardial deformation is based on the arrangement of myocardial architecture. The LV myocardial architecture has an oblique helical fiber arrangement with a right-handed helix in the subendocardial region that gradually changes into a left-handed helix in the subepicardial region. Myocardial fibers of the LV consist of endo- and epicardial layers composed of longitudinal fibers and mid-myocardial layers formed by circumferential fibers. In systole, the shortening of longitudinal fibers causes the displacement of the LV basal plane towards the apex, while the shortening of circumferential fibers induces radial thickening. Deformation in both of these planes reduces LV during systole [35–38]. Accordingly, LVEF is the result of both longitudinal and circumferential fibers but is unable to distinguish functional impairment of one of these components. Longitudinal function impairment can precede the reduction in circumferential indices, giving rise to subclinical impairment of LV pump function [39–42].

Technological advance made possible the assessment of myocardial deformation in different planes corresponding to LV fiber orientation, and several echocardiographic methods have been applied.

Doppler myocardial imaging (or tissue Doppler imaging (TDI)) examines the longitudinal component of myocardial contraction throughout the cardiac cycle. TDI measurements are more sensitive than conventional echocardiography for detecting early myocardial alterations [43, 44]. However, TDI is affected by angle dependency and is not  suitable for assessment of deformation in the circumferential and radial directions as well as rotation. In addition, the measurement of myocardial velocities is influenced by cardiac translational artifacts.

The more recent method of two- and three-dimensional speckle tracking echocardiography (STE) has completely revolutionized the myocardial deformation imaging field providing an estimation of myocardial deformation by measuring strain and strain rate. Strain is described as a deformation of the myocardium during the cardiac cycle in the longitudinal, circumferential, and radial planes. Strain is defined as the change in length of a myocardial segment relative to its resting length while a strain rate is defined as the rate of such deformation [45].

Evidences demonstrated that global longitudinal strain (GLS) can be more sensitive to detect LV impairment than LVEF providing better insight into myocardial impairment. GLS can be altered in patients with HFpEF suggesting unrecognized myocardial systolic dysfunction and can be associated with worse clinical outcomes [41, 46, 47].

Speckle tracking-based deformation analysis predominantly relies on semi-automatic image segmentation (delineation) and quantification techniques, providing a reproducible platform. Different analysis algorithms from different vendors may affect the reproducibility among different echocardiography; however, recent standardization should improve the robustness of the technique [45].

Despite the fact that echocardiography is the first-line imaging method in the workup of HF patients, however CMR has emerged as an indispensable diagnostic tool over the last few years.

CMR provides at the same time information on chamber morphology, dimension and systolic function, myocardial perfusion, valve anatomy and function, and vessel and tissue characterization. Several sequences are available, and they can be combined in different protocols in order to answer to a clinical question [48].

CMR is considered the best alternative imaging modality to provide accurate and reproducible measurements on bi-ventricular dimension and systolic function in patients with poor acoustic window or doubtful echocardiogram (class I evidence C) [5] due to excellent contrast of soft tissues to characterize myocardial structure and function. The quantification of LVEF, volume and mass, is accurate and reproducible particularly in hearts geometrically distorted. CMR calculate LV volumes by summating multiple equally spaced slices in end-diastole or end-systole, requiring no geometric assumptions.

However, the added value of the technique is the possibility to non-invasively tissue characterization of the myocardium. CMR allows the detection of myocardial focal abnormalities, such as edema, fat, iron overload, and fibrosis. Particularly the late gadolinium enhancement (LGE) technique represents the keystone of tissue characterization and provides insights into the underlying causes of myopathy, distinguishing between ischemic and non-ischemic etiologies. The presence, the distribution, and the extent of LGE provide information on the underlying etiology to exclude conditions with phenotypic overlap and, importantly, to exclude ischemic heart disease as a potentially reversible cause.

Patients with an ischemic etiology of LV dysfunction demonstrate subendocardial or transmural LGE in a coronary artery distribution whereas those with non-ischemic causes have either no LGE or LGE with a non-ischemic pattern. A non-ischemic pattern can be mid-wall, subepicardial, or patchy. LGE is a robust, validated technique and its presence carries important prognostic information [49–53]. However, a gap remained: the LGE technique could not detect global myocardial changes such as those occurring in diffuse fibrosis [54] or in some clinical patterns of myocarditis [55]. There can be large amounts of diffuse fibrosis outside the area of LGE (called “remote” myocardium). Indeed, myocardial fibrosis exists as a continuous spectrum between focal and diffuse fibrosis. It is in this gap that the T1 mapping technique provides new information.

T1 mapping sequence is acquired in a single breath-hold. Within the T1 map, each given pixel value directly corresponds to its underlying T1 relaxation time that can be seen in color and formally quantified. T1 mapping is tissue-specific and increased in the presence of edema, fibrosis, and amyloid, while it is reduced in the case of iron overload or fat presence [56]. In clinical practice and research, there are 2 ways to use T1 mapping: before contrast (native T1 mapping) and with contrast, to generate the extracellular volume fraction (ECV). Acquiring the T1 map before and after contrast administration, one can possibly calculate the ECV [57], representing the space between cells in the myocardium. The formula to calculate ECV is ECV = (1 – hematocrit) × *λ* [58], where *λ* is the partition coefficient. The myocardial intracellular volume (ICV) is calculated as 1 − ECV. This technique has been validated in against histology in several cardiac diseases [58–63], and a normal reference range has been described. T1 mapping represents a new era of tissue characterization allowing seeing and measuring diffuse processes and non-invasively dichotomizing the myocardium in its cellular and extracellular component. Limitations exist mainly related to the different values obtained by different vendors and different sequences; however, recent consensus should increase the robustness of the technique [57].

T1 mapping holds the potential to detect myocardial changes early and carries prognostic information [64]. Recently an imaging protocol including echocardiography and CMR for differentiating hypertensive heart disease and HFpEF found that both GLS and ECV are able to independently discriminate between hypertensive heart disease and HFpEF and identify patients with prognostically significant functional limitation [65]. Furthermore, diffuse myocardial fibrosis by T1 mapping independently predicts invasively measured LV stiffness in HFpEF [66].

### Heart failure classification beyond ejection fraction

In the complexity of heart failure syndrome, identification of pathophysiology and etiology is the only way to define a correct diagnosis. Additionally, physicians must be reminded that management of HF is not just taking care of its symptoms. It is mandatory to decipher the mechanisms underlying HF that is a multiorgan syndrome. Taking into consideration the involvement of the systemic organ is the key for success. Recently, we proposed a new staging system for HF, named HLM (A-B) [67, 68], in analogy with TNM classification used in oncology: “H” for heart damage, which may be analogous with “T” of tumor; lung involvement (L), since for the functional and anatomic proximity of the lungs to the heart, they may be considered lymph node station of the heart, in analogy with “L” of TNM; and malfunction (M) of peripheral organs such as the kidney, liver, brain, and hematopoietic system, taking in mind the etymological meaning of the term metastasis: “what is beyond there.” Each parameter is allocated in four levels of severity (H1–H4, L0–L3, M0–M3).

HLM classification integrates clinical, laboratory, and instrumental parameters concerning the heart, lungs, and other organs. In fact, using the integration of multiple variables, HLM is aimed at going beyond the simple consideration of only the cardiac performance or the mere LVEF value or the heart-lung axis alone, operated by the most common classifications.

Therefore, it is important to decipher pathophysiological mechanisms that underlie the heart damage [69–71]; also, it is fundamental to identify any involvement of systemic organs [72–75]. In the management of patients with HF, instead of utilizing the new ESC classification of preserved, mid-range, and reduced LVEF to identify HF patients, we propose an alternate approach by HLM classification and multimodality imaging.

## Conclusion

Our review underlines the pitfalls of the evaluation of LVEF by two-dimensional echocardiography and shows the need to go beyond its measure in daily management of HF patients. In order to reach the best stratification of the patient and to choose the most appropriate treatment, it is fundamental to comprehend the pathophysiological mechanisms and structural and functional modifications underlying HF; also, it is essential to change our cardiocentric perspective into a holistic point of view of heart failure that, in analogy with a cancer, involves other organs such as the lungs, kidney, and liver, reducing prognosis, often independent of symptoms and LVEF value.
