# Correction to: Structural and myocardial dysfunction in heart failure beyond ejection fraction

**DOI:** 10.1007/s10741-019-09845-7

**Published:** 2019-08-24

**Authors:** Paolo Severino, Viviana Maestrini, Marco Valerio Mariani, Lucia Ilaria Birtolo, Rossana Scarpati, Massimo Mancone, Francesco Fedele

**Affiliations:** Department of Cardiovascular, Respiratory, Nephrology, Anesthesiology and Geriatric Sciences, Sapienza University of Rome, Rome, Italy

**Correction to: Heart Fail Rev**

10.1007/s10741-019-09828-8

The original version of this article unfortunately contained a mistake. Unfortunately one of the author’s last name is misspelled: the correct name is Viviana Maestrini. The original article has been corrected.

